# Pulmonary cryptococcosis induces chitinase in the rat

**DOI:** 10.1186/1465-9921-9-40

**Published:** 2008-05-15

**Authors:** Alfin G Vicencio, Swati Narain, Zhongfang Du, Wang Yong Zeng, James Ritch, Arturo Casadevall, David L Goldman

**Affiliations:** 1 Department of Pediatrics, Division of Respiratory and Sleep Medicine, Albert Einstein College of Medicine and Children's Hospital at Montefiore, Bronx, USA; 2 Department of Pediatrics, Division of Infectious Diseases, Albert Einstein College of Medicine and Children's Hospital at Montefiore, Bronx, USA; 3 Department of Microbiology and Immunology, Albert Einstein College of Medicine, Bronx, USA

## Abstract

**Background:**

We previously demonstrated that chronic pulmonary infection with *Cryptococcus neoformans *results in enhanced allergic inflammation and airway hyperreactivity in a rat model. Because the cell wall of *C. neoformans *consists of chitin, and since acidic mammalian chitinase (AMCase) has recently been implicated as a novel mediator of asthma, we sought to determine whether such infection induces chitinase activity and expression of AMCase in the rat.

**Methods:**

We utilized a previously-established model of chronic *C. neoformans *pulmonary infection in the rat to analyze the activity, expression and localization of AMCase.

**Results:**

Our studies indicate that intratracheal inoculation of *C. neoformans *induces chitinase activity within the lung and bronchoalveolar lavage fluid of infected rats. Chitinase activity is also elicited by pulmonary infection with other fungi (e.g. *C. albicans*), but not by the inoculation of dead organisms. Enhanced chitinase activity reflects increased AMCase expression by airway epithelial cells and alveolar macrophages. Systemic cryptococcosis is not associated with increased pulmonary chitinase activity or AMCase expression.

**Conclusion:**

Our findings indicate a possible link between respiratory fungal infections, including *C. neoformans*, and asthma through the induction of AMCase.

## Background

Asthma is the most common chronic illness in childhood, and rates of disease are highest in urban areas including the Bronx. Fungal infections including *Aspergillus fumigatus *and dermatophytic infections can exacerbate asthma symptoms in certain patients [1,2]. Previous studies from our laboratory demonstrate that sub-clinical pulmonary infection with *Cryptococcus neoformans*, a fungus present in high concentrations in pigeon guano, induces increased airway reactivity in a rat model [3]. Interestingly, we have also demonstrated that sub-clinical *C. neoformans *infection is common among Bronx children, a cohort with an extraordinarily high rate of asthma, suggesting a potential role for this type of infection in asthma pathogenesis [4]. Nonetheless, the mechanisms by which chronic infection by *C. neoformans *and other fungal organisms could lead to asthma are unknown.

Acidic mammalian chitinase (AMCase) has emerged as an important mediator of allergic asthma in both animal models and in humans [5-7]. Chitin, the 2^nd ^most abundant polysaccharide in nature and the substrate of AMCase, is found in fungal cell walls, the exoskeletons of insects and crustaceans, and parasitic nematodes, prompting some investigators to hypothesize that mammalian chitinase might contribute to the pathogenesis of allergic immune responses including asthma. Evidence from their laboratory demonstrates that over-expression of IL-13 induces endogenous AMCase activity and results in airway hyperreactivity [5], and that blockage of AMCase activity attenuates the response. Additional investigators have linked human asthma to specific polymorphisms of AMCase [7]. In the current study, we sought to determine whether pulmonary cryptococcosis induces endogenous chitinase activity and AMCase expression in the rat.

## Methods

### Animal model

We utilized our previously-established protocol to induce chronic infection [3]. Briefly, male Fischer 344 rats weighing 200–250 grams (6–8 weeks of age) were obtained form Taconic Farms (Germantown, NY). Rats were infected intratracheally or intraperitoneally with either 1 × 10^7^yeast or PBS as a control. Studies were also done with Brown Norway and Sprague Dawley rats. In some experiments, *C. neoformans *was killed in 70°C for 30 minutes prior to inoculation. All animal work was carried out with the approval of the Animal Use Committee at the Albert Einstein College of Medicine.

### Yeast

Based on previous experiments, *C. neoformans*, American Type Culture Collection (ATCC) strain 24067 was used to establish infection [8]. In addition, *C. albicans *ATCC strain 200498 was used in certain experiments. Yeasts were grown in Sabouraud's broth at 30°C for 2 days, washed with PBS and counted using a hemacytometer prior to inoculation.

### Bronchoalveolar lavage (BAL) and processing of lung tissue

At different times following inoculation, rats were killed by inhalational exposure to CO_2_. Tracheas were cannulated with a 16 G angiocath and lungs lavaged with 3 ml of PBS four times. Following BAL, the right lung was removed, homogenized in 5 ml of PBS, centrifuged and frozen in -80°C until analysis.

### Fungal Burden

Lung homogenates and BAL fluid (BALF) were inoculated on Sabouraud's agar plates and incubated at 37°C for 72 hours. Colonies were then enumerated. Fungal burdens were log_10 _converted and averaged. For the lungs, fungal burden was expressed per lung. For BAL, fungal burden was expressed per ml.

### Chitinase activity assay

Chitinase activity assay was performed utilizing an established protocol [5]. Briefly, BALF and whole lung homogenates were incubated with 4-methylumbelliferyl-D-N,N'-diacetylchitobioside (Sigma), which is cleaved by bioactive chitinase, thereby releasing a quantifiable fluorogenic substance, 4-methylumbelliferone (excitation 350 nm; emission 450 nm). Bioactive chitinase from *Serratia marcescens *(Sigma) was used for standard measurements. In some experiments, chitinase activity in the supernatant of *C. neoformans *cultures was measured.

### Western blot analysis

BALF and lung homogenates from animals were pooled by infection status and time of infection. Ten microliters of the combined samples were separated with a 10% polyacrylamide gels under denaturing conditions and transferred to nitrocellulose. Western blots were performed with a rabbit polyclonal antibody against AMCase (1:500, a generous gift from Dr. Jack Elias, Yale University) and GAPDH (1:2000, Santa Cruz Biotechnology, Santa Cruz, CA). The secondary antibody for both primary antibodies was a peroxidase-labeled goat anti-rabbit IgG (Southern Biotechnology Associates, Birmingham, AL) at a dilution of 1: 2500 for GAPDH detection and 1:5000 for AMCase detection. Bands were visualized using enhanced chemiluminescense according to the manufacturer's instructions (SuperSignal, Pierce Chemicals, Rockford, IL). Reactivity at 50 kDa, the approximate molecular weight of AMCase, was used to indicate AMCase expression [9].

### Densitometric analysis

Densitometry measurements of western blots were obtained with Image J software (NIH), and values were normalized to GAPDH. Results were reported in arbitrary units.

### Immunohistochemistry

Lungs were fixed in formalin, embedded in paraffin, and subsequently processed for immunohistochemical analysis as described previously [10]. Briefly, 5 μm sections were deparaffinized in xylene and rehydrated in graded concentrations of ethanol. Antigen retrieval was performed in a pressure cooker using 6.5 mM sodium citrate (pH 6.0). Sections were incubated with rabbit polyclonal antibody directed against AMCase (1:100, Dr. Jack Elias, Yale). Staining was visualized utilizing a commercially available kit (Santa Cruz). Negative controls were stained simultaneously with omission of primary antibody.

### Statistical analysis

Student's T-test was used to compare individual differences in chitinase activity between infected and control animals. For multiple comparisons, one-way analysis of variance testing was used. Post-hoc testing was done using the Dunnett test. Differences between groups were considered significant at *p *< 0.05.

## Results

### Fungal burden

All rats inoculated with *C. neoformans *and *C. albicans *were actively infected at the time of chitinase determinations. Two days following intratacheal inoculation average log_10 _fungal burdens in BAL (per ml) and lung (per entire right lung) were 4.42 ± 0.01 and 6.48 ± 0.01, respectively Fourteen days following intratracheal inoculation the average log_10 _fungal burdens in BAL and lung were as follows: 3.97 ± 0.19 and 6.33 ± 0.03. At 2 months, the average lung fungal burden was log_10 _5.4 ± 0.02 (BAL was not tested). At 2 days following intraperitoneal inoculation the average log_10 _fungal burden in the lung was 4.5 ± 0.04, however the fungal burden in the BAL was below the limits of detection (log_10 _2). For rats intratracheally infected with *Candida albicans *the log_10 _fungal burden lung was 4.39 ± 0.06. No fungus was detected in the BAL limits of detection log_10 _2. Fungus was not detected in rats inoculated with heat-killed organisms.

### Chitinase activity

**Figure 1:**
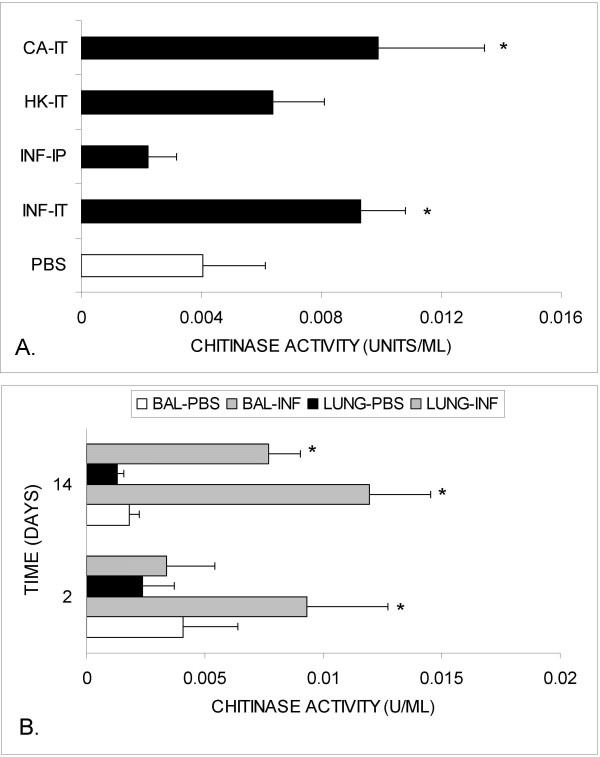
**Early chitinase activity in BALF and whole lung (Fisher rats)**. **A) **Two days following inoculation, chitinase activity was increased in BALF from rats intratracheally infected with *C. neoformans *(INF-IT) and *C. albicans *(CA-IT) when compared to controls. Rats inoculated intraperitoneally with *C. neoformans *(INF-IP) did not manifest an increase in BALF chitinase activity. Rats inoculated intratracheally with heat-killed *C. neoformans *(HK-IT) did not demonstrate statistically significant increase in BALF chitinase activity. **B) **Chitinase activity remained elevated in the BALF on day 14 of infection (BAL-INF) when compared with controls (BAL-PBS). In addition, an increase in whole lung chitinase activity was noted in infected rats (LUNG-INF; n = 4–5 rats/group; *p-value < 0.05 vs. controls).

As demonstrated in Figure 1A, a >2-fold increase in BALF chitinase activity was present as early as 2 days after intratracheal inoculation of *C. neoformans*. Similar chitinase activity was also present in the BALF of rats inoculated with *C. albicans*. Although chitinase activity appeared to be increased in the BALF of rats inoculated with heat killed *C. neoformans *on day 2, this increase was not statistically significant. Intraperitoneal inoculation of *C. neoformans *did not result in increased chitinase activity in BALF. No differences in chitinase activity were detected in whole lung homogenates on day 2 (Figure 1B).

**Figure 2:**
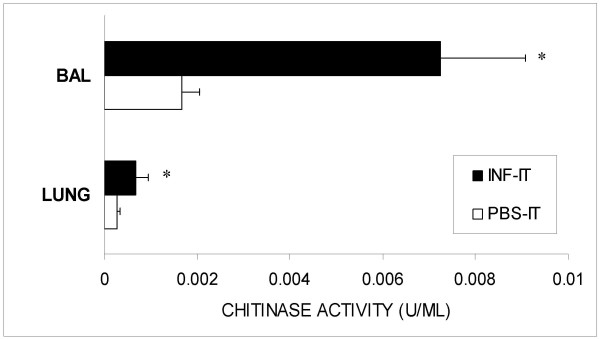
**Persistent chitinase activity in BALF and whole lung**. Two months following intratracheal infection with *C. neoformans*, chitinase activity remained increased in both BALF and whole lung homogenate from intratracheally infected animals compared to controls (n = 4–5 rats/group; *p-value < 0.05 vs. controls).

Interestingly, 14 days after single intratracheal inoculation with *C. neoformans*, both BALF and whole lung chitinase activity from infected animals increased to >6-fold compared to controls (Figure 1B). In contrast, chitinase activity was not detected in either BALF or lung homogenates 14 days after inoculation with heat-killed *C. neoformans*. At 2-months of infection, chitinase activity remained elevated in BALF (3.5-fold) and whole lung homogenates (>2-fold) relative to PBS-inoculated animals (Figure 2). To insure that the observed chitinase activity was not due to fungal expression of chitinase, we tested cryptococcal culture supernatatants at multiple times following inoculation (days 1, 3, and 7). The density of organisms in culture by day 3 was approximately log_10 _7.30 ± 0.04 per ml. No chitinase activity was detected.

We repeated experiments with Brown Norway and Sprague Dawley rats because of their known tendencies to exhibit different degrees of TH2 polarization and allergic inflammation in response to antigenic stimulation [11,12]. At 2 weeks, chitinase activity was increased in the both the BAL and lung homogenate of infected rats relative to PBS-inoculated animals, regardless of rat strain (Table 1).

**Table 1:** Chitinase expression in three different strains of rats infected with *C. neoformans*

	TH bias**	BAL		LUNG	
		PBS	INFECTED	PBS	INFECTED
Fischer	1 [3,11]	1.8 ± 0.4	11.9 ± 2.6*	2.1 ± 0.3	10.6 ± 1.3*
Sprague Dawley	Outbred, Non-biased [12,20]	1.8 ± 0.5	15.2 ± 4.4*	0.7 ± .01	6.4 ± 1.0*
Brown Norway	2 [12,20]	1.9 ± 0.5	6.6 ± 3.9*	1.4 ± .03	5.1 ± 1.7*

Chitinase activity (units × 10^-3^/ml) in different rat strains following experimental pulmonary cryptococcal infection. Values represent means ± 1 SD. For all experiments 4–5 animals were used per group and measurements done at 2 weeks. * P < 0.05 for comparison between infected tissues and PBS-inoculated controls. **TH bias of rat strain reflects tendency to develop TH1 (cellular) versus TH2 (humoral/allergic) inflammation in response to infection or antigen challenge.

### AMCase protein expression

**Figure 3:**
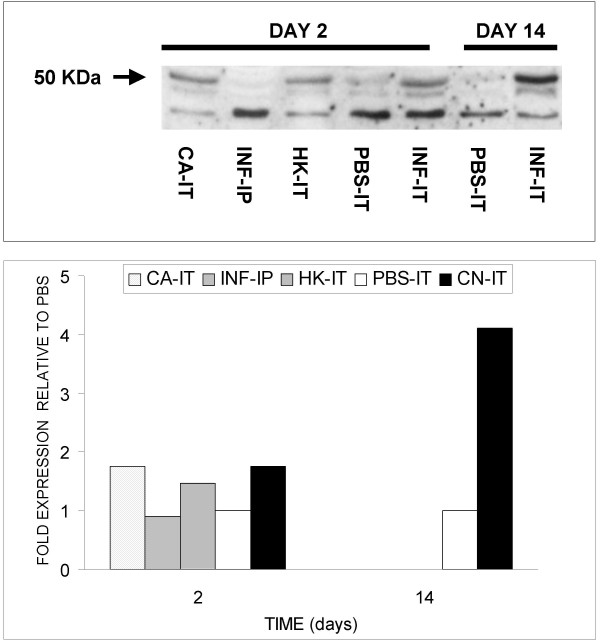
**AMCase expression in BALF**. Western blot analysis and densitometry of BALF demonstrated that AMCase expression parallels general chitinase activity. CA-IT, INF-IT, HK-IT and PBS-IT refer to rats intratracheally inoculated with *C. albicans*, *C. neoformans*, heat-killed *C. neoformans *and PBS respectively (each lane = pooled sample of 6 animals). For densitometry analyses, readings were normalized to GAPDH signal prior to comparison with values obtained from PBS-inoculated animals.

Because we observed significant increases in generalized chitinase activity in both BALF and whole lung from infected rats, and since several chitinase family members exhibit enzymatic activity, we sought to determine whether the specific expression of AMCase was induced by *C. neoformans *infection. As demonstrated in Figure 3, the pattern of AMCase expression paralleled chitinase activity. Specifically, increased AMCase expression was elevated in BALF as early as 2 days following intratracheal inoculation with both live *C. neoformans *(1.8-fold) and *C. albicans *(1.8-fold) compared with controls. There was no observable difference in AMCase expression with systemic infection. Also consistent with chitinase activity data, the increased AMCase expression in BALF persisted at day 14 (4-fold increase). Lastly, AMCase expression was not detected in lung homogenates at Day 2, but was clearly increased at day 14 compared to controls (7.3-fold corrected for GAPDH, data not shown).

### Localization of AMCase expression

**Figure 4:**
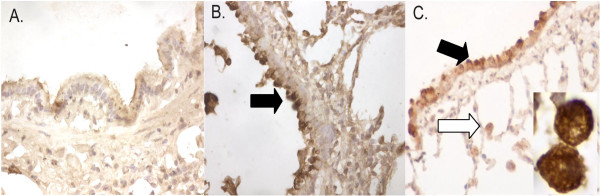
**Immunostaining for AMCase expression**. **A) **Immunostaining for AMCase expression revealed minimal reactivity in the lungs of controls. **B) **Intense signal was demonstrated in airway epithelium (black arrow), particularly after 2 months of infection. **C) **Similar expression patterns in the airway epithelium (black arrow) and alveolar macrophages (white arrow) were also seen at 2 weeks. Original magnification 200× for all photomicrographs. Inset shows high magnification (original magnification 1000×) of immunoreactive cells that appear to be alveolar macrophages.

As demonstrated in Figure 4, control animals demonstrated little signal in airway epithelium. In comparison, AMCase expression was seen at day 14 and at 2-months in airway epithelium (black arrows) and alveolar macrophages (white arrows).

## Conclusion

To our knowledge, ours is the first investigation to demonstrate that intratracheal infection with *C. neoformans *induces generalized chitinase activity in BALF and lung homogenate from rats. Furthermore, we describe that this activity persists with chronic pulmonary infection, but is not present with systemic infection. We also demonstrate that increased AMCase expression in airway epithelial cells and alveolar macrophages parallels generalized chitinase activity and is consistent with previous investigations [5,13].

*C. neoformans *is well known for its tendency to elicit allergic inflammation in animal models [14]. In previous studies, we have shown that pulmonary cryptococcosis not only elicits allergic inflammation, but also promotes both allergic inflammation in response to other antigen exposures and non-specific airway responsiveness [3]. The pathogen specific factors that lead to allergic inflammation are not well understood, but several investigators have focused on the role of the cryptococcal polysaccharide [15,16].

Our study suggests that induction of AMCase by *C. neoformans *is another mechanism that may promote allergic inflammation. Importantly, additional proteins with chitinase activity have also been discovered in both rodents and humans, including BRP-39 (mouse), YKL-40 (human) and chitotriosidase [17]. While our investigation suggests that the increase in generalized chitinase activity in the rat is partly due to over expression of AMCase, we are unable to determine whether BRP-39 and chitotriosidase are similarly upregulated.

Chitin is a major constituent of the fungal cell wall so it is not surprising that induction of chitinase and AMCase were also observed with *C. albicans*. Interestingly, sustained induction of chitinase activity was not observed with inoculation of heat-killed organisms suggesting that active infection is required for chitinase and AMCase induction. It is not clear whether this difference in chitinase induction reflects a qualitative or quantitative difference in exposure. Future studies will focus on the role of infection-induced epithelial damage in promoting chitinase expression. *C. neoformans *is one of several fungi including that is capable of cause persistent sub-clinical pulmonary disease. We hypothesize that these types of fungal infections have the potential of modifying the cytokine milieu within the lung thereby promoting allergic responses to subsequent antigen exposures.

Although the current study demonstrates chitinase induction by *C. neoformans *in a rat model, similar mechanisms could contribute to the high rates of human asthma in urban areas such as the Bronx. As mentioned, our past studies demonstrated a high incidence of sub-clinical infection in Bronx children well beyond that seen in other areas [18]. It is interesting that such infection parallels the incidence of asthma. We also note that chitin is also major constituent of other exposures that have been linked with urban asthma including cockroaches [19]. Further investigation in human populations will be needed to determine the significance chitinase-like protein induction in asthma pathogenesis.

## Competing interests

The authors declare that they have no competing interests.

## Authors' contributions

AGV, oversaw development of immunostaining and immunoblot. He was also directly involved in preparation of manuscript, SN carried out chitinase assay standardization and measurements, ZD carried out immunostaining studies, WYZ infected animals and carried out chitinase and immunoblot assays. JR assisting in immunostaining and processing of tissues. AC assisted in manuscript preparation and development of project. DLG developed project, assisted in manuscript preparation and was responsible for oversight of entire project.

## References

[B1] Ward GW, Woodfolk JA, Hayden ML, Jackson S, Platts-Mills TA (1999). Treatment of late-onset asthma with fluconazole. J Allergy Clin Immunol.

[B2] Wark PA, Gibson PG, Wilson AJ (2004). Azoles for allergic bronchopulmonary aspergillosis associated with asthma. Cochrane Database Syst Rev.

[B3] Goldman DL, Davis J, Bommarito F, Shao X, Casadevall A (2006). Enhanced allergic inflammation and airway responsiveness in rats with chronic Cryptococcus neoformans infection: potential role for fungal pulmonary infection in the pathogenesis of asthma. J Infect Dis.

[B4] Goldman DL, Khine H, Abadi J, Lindenberg DJ, Pirofski L, Niang R, Casadevall A (2001). Serologic evidence for Cryptococcus neoformans infection in early childhood. Pediatrics.

[B5] Zhu Z, Zheng T, Homer RJ, Kim YK, Chen NY, Cohn L, Hamid Q, Elias JA (2004). Acidic mammalian chitinase in asthmatic Th2 inflammation and IL-13 pathway activation. Science.

[B6] Zhao J, Zhu H, Wong CH, Leung KY, Wong WS (2005). Increased lungkine and chitinase levels in allergic airway inflammation: a proteomics approach. Proteomics.

[B7] Bierbaum S, Nickel R, Koch A, Lau S, Deichmann KA, Wahn U, Superti-Furga A, Heinzmann A (2005). Polymorphisms and haplotypes of acid mammalian chitinase are associated with bronchial asthma. Am J Respir Crit Care Med.

[B8] Goldman D, Lee SC, Casadevall A (1994). Pathogenesis of pulmonary Cryptococcus neoformans infection in the rat. Infect Immun.

[B9] Boot RG, Blommaart EF, Swart E, Ghauharali-van der Vlugt K, Bijl N, Moe C, Place A, Aerts JM (2001). Identification of a novel acidic mammalian chitinase distinct from chitotriosidase. J Biol Chem.

[B10] Goldman D, Cho Y, Zhao M, Casadevall A, Lee SC (1996). Expression of inducible nitric oxide synthase in rat pulmonary Cryptococcus neoformans granulomas. Am J Pathol.

[B11] Ohtsuka R, Shutoh Y, Fujie H, Yamaguchi S, Takeda M, Harada T, Doi K (2005). Changes in histology and expression of cytokines and chemokines in the rat lung following exposure to ovalbumin. Exp Toxicol Pathol.

[B12] Hylkema MN, Hoekstra MO, Luinge M, Timens W (2002). The strength of the OVA-induced airway inflammation in rats is strain dependent. Clin Exp Immunol.

[B13] Boot RG, Bussink AP, Verhoek M, de Boer PA, Moorman AF, Aerts JM (2005). Marked differences in tissue-specific expression of chitinases in mouse and man. J Histochem Cytochem.

[B14] Hernandez Y, Arora S, Erb-Downward JR, McDonald RA, Toews GB, Huffnagle GB (2005). Distinct roles for IL-4 and IL-10 in regulating T2 immunity during allergic bronchopulmonary mycosis. J Immunol.

[B15] Almeida GM, Andrade RM, Bento CA (2001). The capsular polysaccharides of Cryptococcus neoformans activate normal CD4(+) T cells in a dominant Th2 pattern. J Immunol.

[B16] Vecchiarelli A, Retini C, Monari C, Tascini C, Bistoni F, Kozel TR (1996). Purified capsular polysaccharide of Cryptococcus neoformans induces interleukin-10 secretion by human monocytes. Infect Immun.

[B17] Chupp GL, Lee CG, Jarjour N, Shim YM, Holm CT, He S, Dziura JD, Reed J, Coyle AJ, Kiener P, Cullen M, Grandsaigne M, Dombret MC, Aubier M, Pretolani M, Elias JA (2007). A chitinase-like protein in the lung and circulation of patients with severe asthma. N Engl J Med.

[B18] Davis J, Zheng WY, Glatman-Freedman A, Ng JA, Pagcatipunan MR, Lessin H, Casadevall A, Goldman DL (2007). Serologic evidence for regional differences in pediatric cryptococcal infection. Pediatr Infect Dis J.

[B19] Eggleston PA, Rosenstreich D, Lynn H, Gergen P, Baker D, Kattan M, Mortimer KM, Mitchell H, Ownby D, Slavin R, Malveaux F (1998). Relationship of indoor allergen exposure to skin test sensitivity in inner-city children with asthma. J Allergy Clin Immunol.

[B20] Hylkema MN, Timens W, Luinge M, Van Der WN, Hoekstra MO (2002). The effect of bacillus Calmette-Guerin immunization depends on the genetic predisposition to Th2-type responsiveness. Am J Respir Cell Mol Biol.

